# New criteria for multiple chemical sensitivity based on the Quick Environmental Exposure and Sensitivity Inventory developed in response to rapid changes in ongoing chemical exposures among Japanese

**DOI:** 10.1371/journal.pone.0215144

**Published:** 2019-04-26

**Authors:** Sachiko Hojo, Atsushi Mizukoshi, Kenichi Azuma, Jiro Okumura, Masami Mizuki, Mikio Miyata

**Affiliations:** 1 Institute of Applied Brain Sciences, Waseda University, Tokorozawa, Japan; 2 Shokei Gakuin University, Natori, Japan; 3 Tohoku University Graduate School of Dentistry, Sendai, Japan; 4 Department of Environmental Medicine and Behavioral Science, Kindai University Faculty of Medicine, Osakasayama, Japan; 5 Department of Respiratory Medicine and Allergy, National Hospital Organization, Morioka National Hospital, Morioka, Japan; 6 Soyokaze Allergic Clinic, Tokyo, Japan; Shahjalal University of Science and Technology, BANGLADESH

## Abstract

**Background & objectives:**

The Quick Environmental Exposure and Sensitivity Inventory (QEESI) developed by Miller and Prihoda in the USA is used as a questionnaire for patients with multiple chemical sensitivity (MCS) in >10 countries. We developed a Japanese version of QEESI, assessed its reliability and validity, and defined original cut-off values for screening Japanese patients with MCS in 2003. Our recent study revealed that opportunities for exposure to various chemicals had increased for people in Japan, while subjective symptoms of MCS in patients had increased in severity. In this study, we considered new cut-off values that combined QEESI subscale scores based on the current situation in Japan.

**Methods:**

The questionnaire used was a Japanese version of QEESI. The survey was conducted from 2012 to 2015. Participants were 111 patients with MCS (mean age: 46 ± 20, 81% female) initially diagnosed by physicians, and 444 age- and gender-matched controls not diagnosed with MCS by doctors. The discriminatory validity of QEESI scores of patients and controls were evaluated by logistic regression and receiver operating characteristic analyses when considering interactions of the Masking Index (ongoing chemical exposure). New combined cut-off values were then set.

**Results:**

New combined cut-off values (meeting conditions of Chemical Intolerances ≥ 30, Symptom Severity ≥ 13, and Life Impacts ≥ 17) showed high sensitivity (82.0%) and specificity (94.4%). Using new criteria when considering ongoing chemical exposure, study participants were categorized as: Very suggestive, Somewhat suggestive, Problematic, and Not suggestive. Participants classified as Very suggestive included 25 (5.6%) controls.

**Conclusions:**

We have set new criteria with combined cut-off values based on current Japanese conditions. Such new criteria can be used for screening and as a diagnostic aid for Japanese patients with MCS and suggest approximately 6% of the Japanese general population may be classified as “Very suggestive people with MCS”.

## Introduction

Multiple chemical sensitivity (MCS), also referred to as an environmental intolerance (EI) to chemicals or toxicant-induced loss of tolerance (TILT), is a health disorder that encompasses various undefined symptoms in multiple organs that mainly include the central and autonomic nervous systems. Symptoms normally develop in response to exposure to an extremely small amount of a chemical substance that an ordinary person is usually insensitive to [1–7].

Patients with symptoms of MCS have been found in Europe, the United States, and Japan, with a reported prevalence of from 8 to 33% of the population, depending on the country and survey method used [8–28]. Nevertheless, many characteristics of MCS remain scientifically unexplained, and internationally agreed-upon diagnostic criteria, treatments, and preventive measures have yet to be put in place. To establish these would require that researchers increasingly document the clinical conditions of patients with MCS and share their findings with other international researchers.

The most comprehensive diagnostic criteria for MCS that is applied worldwide is known as “the 1999 consensus, USA” [2, 29]. A recommended questionnaire in “the 1999 consensus” is an environment exposure sensitivity inventory (EESI) and its short version, a quick environment exposure sensitivity inventory (QEESI), which were both developed by Miller and Prihoda [30, 31]. QEESI has been translated into several languages (e.g. Japanese, Swedish, Dutch, Korean, Taiwanese, and Italian) and is used in many countries [8, 16–22, 27, 32–50].

The Japanese version of QEESI was translated by Ishikawa and Miyata [49] while Hojo et al. [35] confirmed its reliability and validity after several modifications to facilitate the answering of questions by a Japanese population. We previously described the original cut-off values applied to a Japanese population [20, 22]. However, we recently revealed that chemical exposures the general Japanese population is subjected to on a daily basis (ongoing chemical exposure) has largely changed and that onset/trigger factors for patients with MCS have become diversified; the subjective symptoms of patients with MCS have become more severe in response to chemical intolerances and life impacts, along with recent acute lifestyle changes for people in Japan [50].

Therefore, in this study, based on such lifestyle changes in Japanese, we considered new cut-off values combining QEESI subscale scores with high sensitivity and specificity using the data of a recent survey conducted between 2012 and 2015 [50], which was available for screening and as a diagnostic aid for suggestive patients with MCS. Additionally, we estimated the proportion of “very suggestive patients with MCS” in the current Japanese population by classifying them according to the new criteria.

## Participants and methods

### Questionnaire

Several survey periods (plural) were held between March 2012 and January 2015 using one questionnaire. The questionnaire included QEESI and additional items related to characteristics necessary for analysis (gender, age, resident prefecture, employment, etc.). In addition to a questionnaire for the general population, an item concerning whether the participant was under treatment for MCS or sick house syndrome (SHS; or sick building syndrome) during the survey was added. The original English version of QEESI has been previously published [30].

QEESI consisted of five subscales, each with 10 items, which in total consisted of 50 items: Q1 Chemical Intolerances, Q2 Other Intolerances, Q3 Symptom Severity, Q4 Masking Index, and Q5 Life Impacts. Four subscales (Q1, Q2, Q3, and Q5) were rated with scores from 0 to 10 for each item. Total scores for Q1, Q2, Q3, and Q5 were between 0 and 100. Subscales of the Q4 Masking Index were aimed at asking whether an ongoing chemical exposure existed, which were then rated by the selection of “yes (1)” or “no (0)” for each item. The total score for Q4 was rated from 0 to 10.

### Participants, distribution and collection of questionnaires

#### Patients with MCS

The patients in this study were diagnosed with MCS when they first visited one of four representative medical institutions that offered specialty outpatient services for people with MCS in Japan. The patient groups in this survey were diagnosed with MCS according to the same diagnostic criteria, satisfying both “the 1999 consensus, USA” [2] and Japanese original diagnostic criteria [49]. In addition to this, patients with a known chronic disease, including lifestyle-related diseases, chronic fatigue syndrome, and fibromyalgia, were excluded. Patients were randomly selected during the survey periods.

In the survey, 111 patients were diagnosed with MCS by four MCS specialist physicians at four medical institutions (Soyokaze Allergic Clinic, Morioka National Hospital, Sagamihara National Hospital, and Kochi National Hospital).

Valid data was that which described age and gender, and showing most QEESI items to have been filled in. The questionnaires were given to 111 patients and valid data was received from all 111 patients (100%).

### Controls

The controls used were a general population living in 35 prefectures of Japan. Controls were recruited through a communication network of various organizations to which the authors of this study belonged such as five academic societies, three research societies, ten universities, three vocational schools, two societies of architects, two regional neighborhood associations, and five environmental non-profit organizations. Each participant filled in the questionnaire anonymously and mailed it directly to the authors. We excluded data from patients diagnosed with SHS and MCS by doctors. Valid data was that which described age and gender, and showing most QEESI items to have been filled in. Questionnaires were send to 2,007 persons, with 1,327 questionnaires returned (participation rate, 66.1%); valid data were derived from 1,313 of these. The controls were 444 randomly selected people matched for age and sex with MCS patients (n = 111) from valid data (n = 1,313).

### Statistical analyses

The internal consistency of four subscales (Q1 Chemical Intolerances, Q2 Other Intolerances, Q3 Symptom Severity, and Q5 Life Impacts) was evaluated using Cronbach’s alpha. *P*-values associated with odds ratios for characteristics (education, occupation, comorbid allergies, and medical history) and the Q4 Masking Index were calculated using two-tailed Fisher's exact test. Odds ratios comparing patients to controls for working hours, and four subscale scores (Q1, Q2, Q3, and Q5) were calculated by univariate analysis and significant differences were evaluated using a Wald test. The items of the Q4 Masking Index were divided into two scales based on odds ratios. Interactions between the divided Masking Index and the other four subscale total scores were evaluated using Spearman’s rank correlation.

The discriminatory validity of QEESI scores for patients and controls was evaluated by logistic regression and receiver operating characteristic (ROC) analyses. Cut-off values with high sensitivity and specificity were calculated for combined selected scales. With regard to interactions of the divided Masking Index, participants were classified into four categories based on their risk for MCS. Data were analyzed using IBM SPSS Statistics, version 22 for Microsoft Windows (IBM, Armonk, NY, USA).

### Ethics considerations

The study was approved by research ethics committees of the Environmental Center of Oita University (No. 304, approved on June 4, 2009), National Hospital Organization (NHO), Morioka National Hospital (No. 24–01, approved on June 6, 2012), and NHO, Sagamihara National Hospital, Sagamihara (No. 6, approved on July 9, 2013), in accordance with the Declaration of Helsinki.

Informed consent for the survey was obtained from all MCS patients who agreed to participate in the survey. The questionnaire was given to patients by an attending physician and informed, written consent was obtained after the study was explained. Each patient filled in the questionnaire anonymously and submitted it to the attending physician. Moreover, the questionnaires were anonymized by each medical institution to prevent individual names being specified and were sent to the person in charge of data entry for the study. For the control group, we sent out questionnaires and return envelopes in response to the number of general people who gave informed consent at each organization.

## Results

### Comparison of characteristics between patients and controls

Table 1 lists the characteristics of patients and controls.

**Table 1:** Characteristics of physician-diagnosed MCS patients and gender- and age-matched controls.

	MCS patients	Controls	Odds ratio	*P*-value^a^
**Female, n (%)**	90 (81.1)	360 (81.1)		1.00
**Male, n (%)**	21 (18.9)	84 (18.9)		
**Total**	111	444		
**Age, mean (SD)**	46.4 (13.8)	46.5 (12.7)		
**Age group, n (%)**				
12–29	9 (8.1)	36 (8.1)		
30–39	23 (20.7)	92 (20.7)		
40–49	40 (36)	157 (35.4)		
50–59	21 (18.9)	87 (19.6)		
60–79	18 (16.2)	72 (16.2)		
**Education, n (%)**				
Primary school	5 (6.4)	7 (1.8)	2.94 (0.92–9.46)	0.070
High school	27 (34.6)	115 (28.8)	0.92 (0.57–1.49)	0.808
College/University	44 (56.4)	248 (62.0)	0.52 (0.34–0.79)	0.003**
Graduate	2 (2.6)	30 (7.5)	0.25 (0.06–1.08)	0.042*
**Working hours, median (IQR)**	8 (7)	9 (3)	0.85 (0.79–0.91)	<0.001***
**Occupation, n (%)**				
Unemployed	30 (29.4)	22 (5.3)	4.47 (2.73–7.31)	<0.001***
Student	5 (4.9)	21 (5.0)	0.95 (0.35–2.58)	1.000
Homeworker	26 (25.5)	66 (15.8)	1.75 (1.05–2.92)	0.045*
Part-time worker	7 (6.9)	67 (16.0)	0.38 (0.17–0.85)	0.013*
Full-time worker	34 (33.3)	242 (57.9)	0.37 (0.24–0.57)	<0.001***
**Comorbid allergies, n (%)**				
Bronchial asthma	17 (16.7)	5 (1.9)	10.6 (3.8–29.6)	<0.001***
Allergic rhinitis	27 (26.5)	11 (4.1)	8.5 (4–17.9)	<0.001***
Allergic conjunctivitis	2 (2.0)	2 (0.7)	2.7 (0.4–19.3)	0.303
Rash	9 (8.8)	2 (0.7)	13.0 (2.8–61.1)	<0.001***
Hay fever	27 (26.5)	16 (5.9)	5.7 (2.9–11.2)	<0.001***
Food allergies	15 (14.7)	1 (0.4)	46.4 (6–356.2)	<0.001***
Other allergic symptoms	25 (24.5)	0 (0.0)	-	
Presence of existing allergy	64 (62.7)	32 (11.9)	12.5 (7.3–21.6)	<0.001***
**Medical history, n (%)**				
Atopic dermatitis	8 (7.8)	26 (9.6)	0.8 (0.3–1.8)	0.690
Bronchial asthma	11 (10.8)	23 (8.5)	1.3 (0.6–2.8)	0.546
Allergic rhinitis	10 (9.8)	48 (17.8)	0.5 (0.2–1)	0.077
Allergic conjunctivitis	8 (7.8)	30 (11.1)	0.7 (0.3–1.5)	0.444
Rash	7 (6.9)	39 (14.4)	0.4 (0.2–1)	0.052
Hay fever	11 (10.8)	55 (20.4)	0.5 (0.2–0.9)	0.033*
Food allergies	4 (3.9)	16 (5.9)	0.6 (0.2–2)	0.608
Other allergic symptoms	9 (8.8)	15 (5.6)	1.6 (0.7–3.9)	0.246
Presence of previous allergy	39 (38.2)	131 (48.5)	0.7 (0.4–1)	0.081

^a^Two-tailed Fisher's exact test for n and Wald test for median; significant at *p < 0.05, **p < 0.01, ***p < 0.001

MCS, multiple chemical sensitivity; SD, standard deviation; IQR, interquartile range

Compared to controls, patients with MCS (1) had a significantly lower proportion of college/university and graduate students (*p* = 0.003 and 0.042, respectively); (2) significantly shorter working hours (*p* < 0.001); and (3) a significantly lower proportion of full-time workers (*p* < 0.001). Compared to controls, patients with MCS (4) had a significantly higher proportion of patients with an existing allergy (*p* < 0.001), with an odds ratio of patients to controls of 12.5; this group also had a significantly higher proportion of unemployed (*p* < 0.001). Regarding each comorbid allergy and compared to controls, patients with MCS showed significant increases for bronchial asthma (odds ratio: 10.6, *p* < 0.001), allergic rhinitis (odds ratio: 8.5, *p* < 0.001), rash (odds ratio: 13.0, *p* < 0.001), hay fever (odds ratio: 5.7, *p* < 0.001), and food allergies (odds ratio: 46.4, *p* < 0.001). Meanwhile, with regard to a medical history of previous allergies, the proportion of patients with MCS having hay fever was significantly lower than that in controls (odds ratio: 0.5, *p* = 0.033); for other prior allergies, a significant difference was not observed.

### Internal consistency in QEESI scores

Cronbach’s alpha for four QEESI subscale total scores are shown in Table 2. High values (≥ 0.8) were observed for both patients and controls except for controls for Q2 Other Intolerances (0.683).

**Table 2:** Cronbach’s alpha for four subscales in QEESI.

	MCS patients	Controls
Q1 Chemical Intolerances	0.953	0.968
Q2 Other Intolerances	0.831	0.683
Q3 Symptom Severity	0.900	0.861
Q5 Life Impacts	0.912	0.845

QEESI, Quick Environmental Exposure and Sensitivity Inventory; MCS, multiple chemical sensitivity

### Comparison of subjective symptoms between patients and controls

#### Frequency distribution

**Fig 1:**
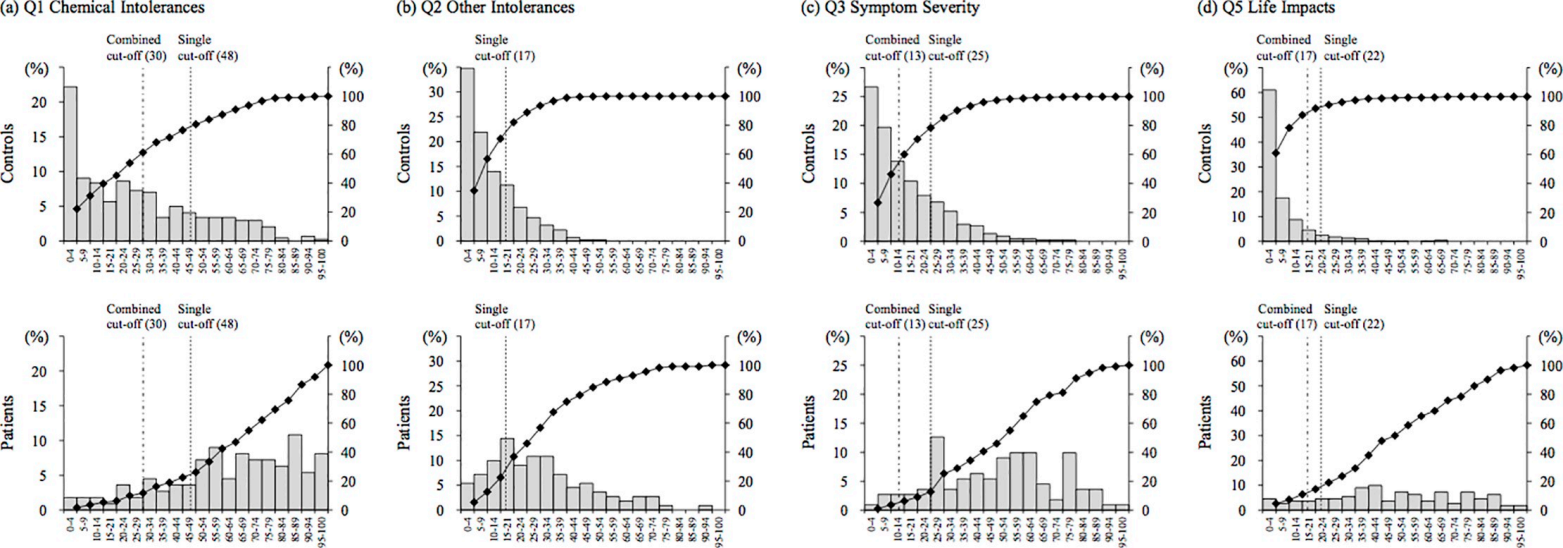
Distribution of total scores of the four scales in QEESI for patients with MCS and controls. QEESI, Quick Environmental Exposure Sensitivity Inventory; MCS, multiple chemical sensitivity.

Frequency distributions for subjective symptoms (total scores of Q1 Chemical Intolerances, Q2 Other Intolerances, Q3 Symptom Severity, and Q5 Life Impacts) are shown in Fig 1. The distributions for patients were broad, including the mode value between medium and high scores for all four scales. Meanwhile, the distributions for controls for Q2, Q3, and Q5 showed a single peak with a mode value between 0 and 4 scores. However, only Q1 Chemical Intolerances showed a broad distribution with medium and high scores.

#### Odds ratios

Table 3 shows the odds ratios of patients to controls for four QEESI subscale scores. Comparing patients to controls, the scores for all items and the total scores for each subscale item were significantly higher (*p* < 0.001 except for q2.6; *p* = 0.008; Table 3).

**Table 3:** Median scores for patients and controls and odds ratios of patients to controls for four subscales in QEESI.

	MCS Patients	Controls	Odds ratio (95% CI)	*P*-value^a^
**Q1 Chemical Intolerances, median (IQR)**				
q1.1 Diesel or gasoline exhaust	6 (4)	2 (4)	1.52 (1.39–1.66)	<0.001***
q1.2 Tobacco smoke	7 (3)	2 (5)	1.43 (1.32–1.55)	<0.001***
q1.3 Insecticide	8 (5)	2 (5)	1.49 (1.38–1.62)	<0.001***
q1.4 Gasoline	6 (4)	2 (5)	1.46 (1.35–1.59)	<0.001***
q1.5 Paint or paint thinner	8 (5)	3 (4)	1.56 (1.43–1.71)	<0.001***
q1.6 Cleaning products	8 (4)	2 (5)	1.66 (1.51–1.82)	<0.001***
q1.7 Fragrances	8 (4)	2 (5)	1.57 (1.44–1.72)	<0.001***
q1.8 Tar or asphalt	6 (6)	2 (4)	1.42 (1.32–1.54)	<0.001***
q1.9 Nail polish or hairspray	7 (5)	2 (4)	1.54 (1.41–1.67)	<0.001***
q1.10 New furnishings	7 (5.5)	1 (4)	1.52 (1.40–1.65)	<0.001***
Total Q1 score	66 (34)	21.5 (37)	1.05 (1.04–1.07)	<0.001***
**Q2 Other Intolerances, median (IQR)**				
q2.1 Chlorinated tap water	3 (5)	0 (2)	1.30 (1.20–1.41)	<0.001***
q2.2 Foods or food additives	4.5 (7)	0 (1)	1.35 (1.26–1.45)	<0.001***
q2.3 Food cravings or feeling ill if meal missed	0 (1)	0 (0)	1.51 (1.28–1.78)	<0.001***
q2.4 Feeling ill after meals	0 (3)	0 (0)	1.50 (1.33–1.70)	<0.001***
q2.5 Caffeine	0 (3.25)	0 (0)	1.40 (1.26–1.55)	<0.001***
q2.6 Feeling ill if caffeine intake is stopped or decreased	0 (0)	0 (0)	1.20 (1.05–1.38)	0.008**
q2.7 Alcohol in small amounts	0 (5.75)	0 (0)	1.24 (1.15–1.33)	<0.001***
q2.8 Fabrics, jewelry, creams and cosmetics that touch the skin	5 (6)	0 (3)	1.39 (1.29–1.50)	<0.001***
q2.9 Adverse reactions to drugs or medications	5 (9)	0 (1)	1.43 (1.33–1.53)	<0.001***
q2.10 Classical allergic reactions	6 (5)	2 (5)	1.28 (1.20–1.38)	<0.001***
Total Q2 score	26 (24)	8 (14)	1.10 (1.08–1.12)	<0.001***
**Q3 Symptom Severity, median (IQR)**				
q3.1 Musculoskeletal	5 (7)	0 (2)	1.48 (1.37–1.60)	<0.001***
q3.2 Airway mucous membranes	6 (4)	1 (3)	1.73 (1.57–1.91)	<0.001***
q3.3 Heart- or chest-related	4 (5)	0 (1)	1.70 (1.54–1.88)	<0.001***
q3.4 Gastrointestinal	5 (6)	1 (2)	1.51 (1.39–1.64)	<0.001***
q3.5 Cognitive	6 (5)	1 (2)	1.71 (1.55–1.89)	<0.001***
q3.6 Affective	5 (6)	1 (2)	1.56 (1.43–1.70)	<0.001***
q3.7 Neuromuscular	6 (6)	1 (2)	1.69 (1.54–1.85)	<0.001***
q3.8 Head-related	7 (5)	0 (2)	1.77 (1.61–1.96)	<0.001***
q3.9 Skin	4 (4)	0 (2)	1.38 (1.28–1.49)	<0.001***
q3.10 Genitourinary	4 (6)	1 (2)	1.40 (1.29–1.51)	<0.001***
Total Q3 score	53 (36)	10 (18)	1.10 (1.08–1.11)	<0.001***
**Q5 Life Impacts, median (IQR)**				
q5.1 Diet	2 (5)	0 (0)	1.77 (1.56–2.01)	<0.001***
q5.2 Ability to work or attend school	7 (8)	0 (0)	1.84 (1.65–2.06)	<0.001***
q5.3 Choice of home furnishings	6 (6.5)	0 (0)	1.86 (1.67–2.07)	<0.001***
q5.4 Choice of clothing	5 (6)	0 (2)	1.59 (1.46–1.73)	<0.001***
q5.5 Ability to drive or travel	6 (6)	0 (0)	1.96 (1.74–2.21)	<0.001***
q5.6 Choice of personal care products	7 (6)	0 (1)	1.65 (1.51–1.79)	<0.001***
q5.7 Ability to be around others and enjoy social activities	7 (5)	0 (0)	2.00 (1.78–2.25)	<0.001***
q5.8 Choice of hobbies or recreation	5 (5)	0 (0)	1.97 (1.75–2.22)	<0.001***
q5.9 Relationships with spouse and family	3 (6)	0 (0)	1.80 (1.59–2.03)	<0.001***
q5.10 Ability to perform household chores	5 (6)	0 (1)	1.83 (1.64–2.05)	<0.001***
Total Q5 score	46 (37)	2 (8)	1.13 (1.10–1.15)	<0.001***

^a^Wald test for median; significant at **p < 0.01, ***p < 0.001

QEESI, Quick Environmental Exposure and Sensitivity Inventory; IQR, interquartile range; CI, confidence interval

### Comparison of Masking Index between patients and controls

#### Frequency distribution

**Fig 2:**
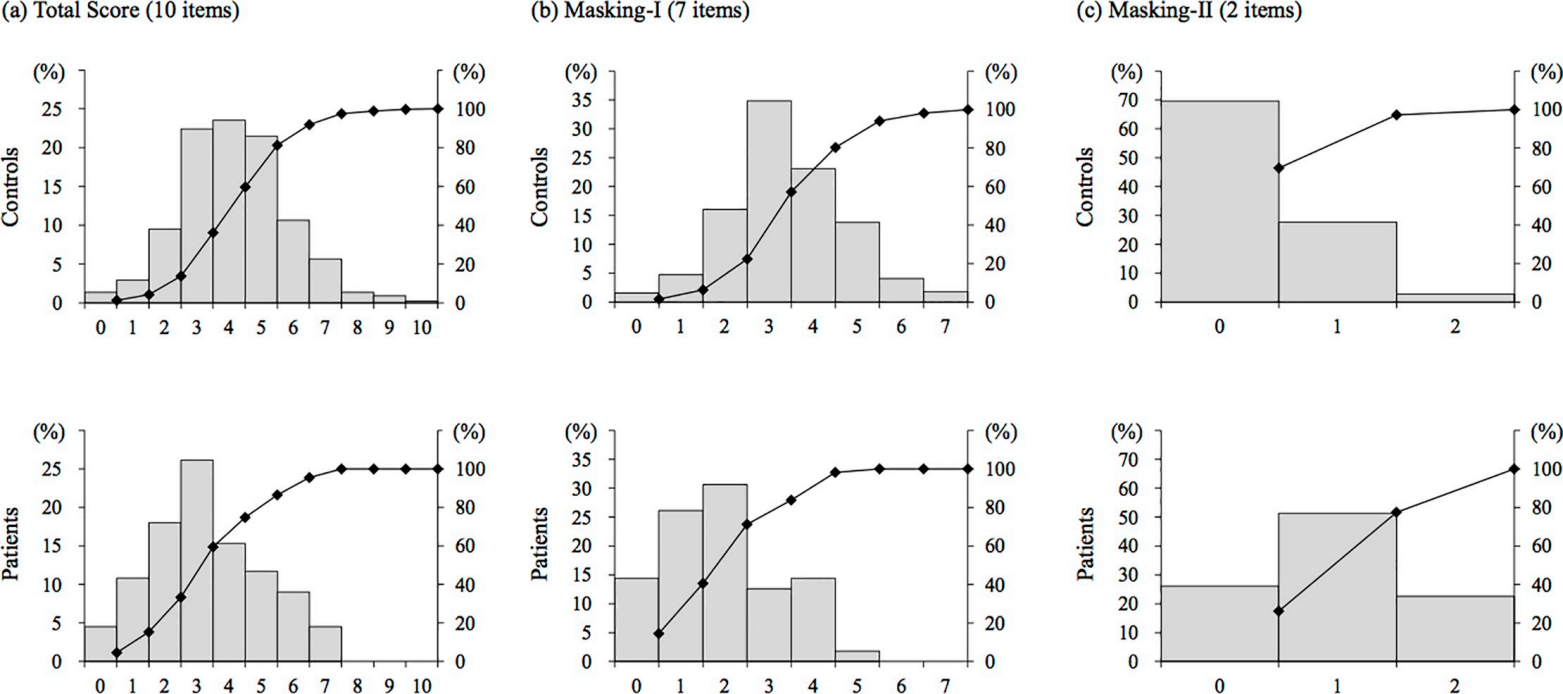
Distribution of total scores of the Q4 Masking Index in QEESI for patients with MCS and controls. a: Total scores, b: Masking-I, c: Masking-II. QEESI, Quick Environmental Exposure Sensitivity Inventory; MCS, multiple chemical sensitivity.

The frequency distribution of the Q4 Masking Index is shown in Fig 2. For controls, a distribution of the total score, including the peak with a mode value between scores of 3 and 5, was observed. For patients, the mode value was a score of 3; however, the proportions of 1 and 2 scores were high compared to controls.

#### Odds ratios

Table 4 shows the odds ratios of patients to controls for each item of the Q4 Masking Index and the total score. Items in Q4 became clearly divided into two groups of items, with odds ratios of less than 1 (items with a lower proportion of patients than controls) and items with odds ratios of more than 1 (items with a higher proportion of patients than controls). Seven items had an odds ratio of less than 1, namely: q4.1 Tobacco (*p* = 0.026), q4.2 Alcohol (*p* < 0.001), q4.3 Caffeine (*p* < 0.001), q4.4 Scented personal care products (*p* < 0.001), q4.7 Second-hand smoke (*p* = 0.027), q4.8 Gas or propane stove (*p* = 0.031), and q4.9 Scented fabric softener (*p* < 0.001); a significant difference was observed for all these items. Meanwhile, three items showed an odds ratio of more than 1, namely: q4.5 Insecticides, q4.6 Chemical or smoke exposure in job or hobby, and q4.10 Drugs; of these, q4.6 (*p* < 0.001) and q4.10 (*p* < 0.001) showed a significant difference.

**Table 4:** Number of patients and controls, and odds ratios of patients to controls, for Q4 Masking Index in QEESI.

Subscale	MCS patients	Controls	Odds ratio (95% CI)	*P*-value^a^
Q4 Masking Index, n (%)				
q4.1 Tobacco	4 (3.7)	45 (10.4)	0.33 (0.12–0.94)	0.026*
q4.2 Alcohol	21 (19.1)	183 (41.8)	0.33 (0.20–0.55)	<0.001***
q4.3 Caffeine	72 (66.1)	364 (82.7)	0.41 (0.25–0.65)	<0.001***
q4.4 Scented personal care products	38 (34.9)	318 (72.1)	0.21 (0.13–0.32)	<0.001***
q4.5 Insecticides	47 (43.5)	188 (42.9)	1.02 (0.67–1.57)	0.914
q4.6 Chemical or smoke exposure in job or hobby	65 (59.6)	56 (12.8)	10.02 (6.24–16.11)	<0.001***
q4.7 Second-hand smoke	15 (13.6)	104 (23.5)	0.51 (0.29–0.92)	0.027*
q4.8 Gas or propane stove	38 (34.9)	205 (46.6)	0.61 (0.40–0.95)	0.031*
q4.9 Scented fabric softener	25 (22.7)	283 (64.2)	0.16 (0.10–0.27)	<0.001***
q4.10 Drugs	42 (38.2)	90 (20.5)	2.40 (1.53–3.76)	<0.001***
Total Q4 score, median (IQR)	3 (3)	4 (2)	0.73 (0.64–0.84)	<0.001***

^a^Two-tailed Fisher's exact test for n and Wald test for median; significant at *p < 0.05, ***p < 0.001

QEESI, Quick Environmental Exposure and Sensitivity Inventory; CI, confidence interval; IQR, interquartile range; MCS, Multiple chemical sensitivity.

### Correlation between Masking Index and each of four subscale total scores

**Fig 3:**
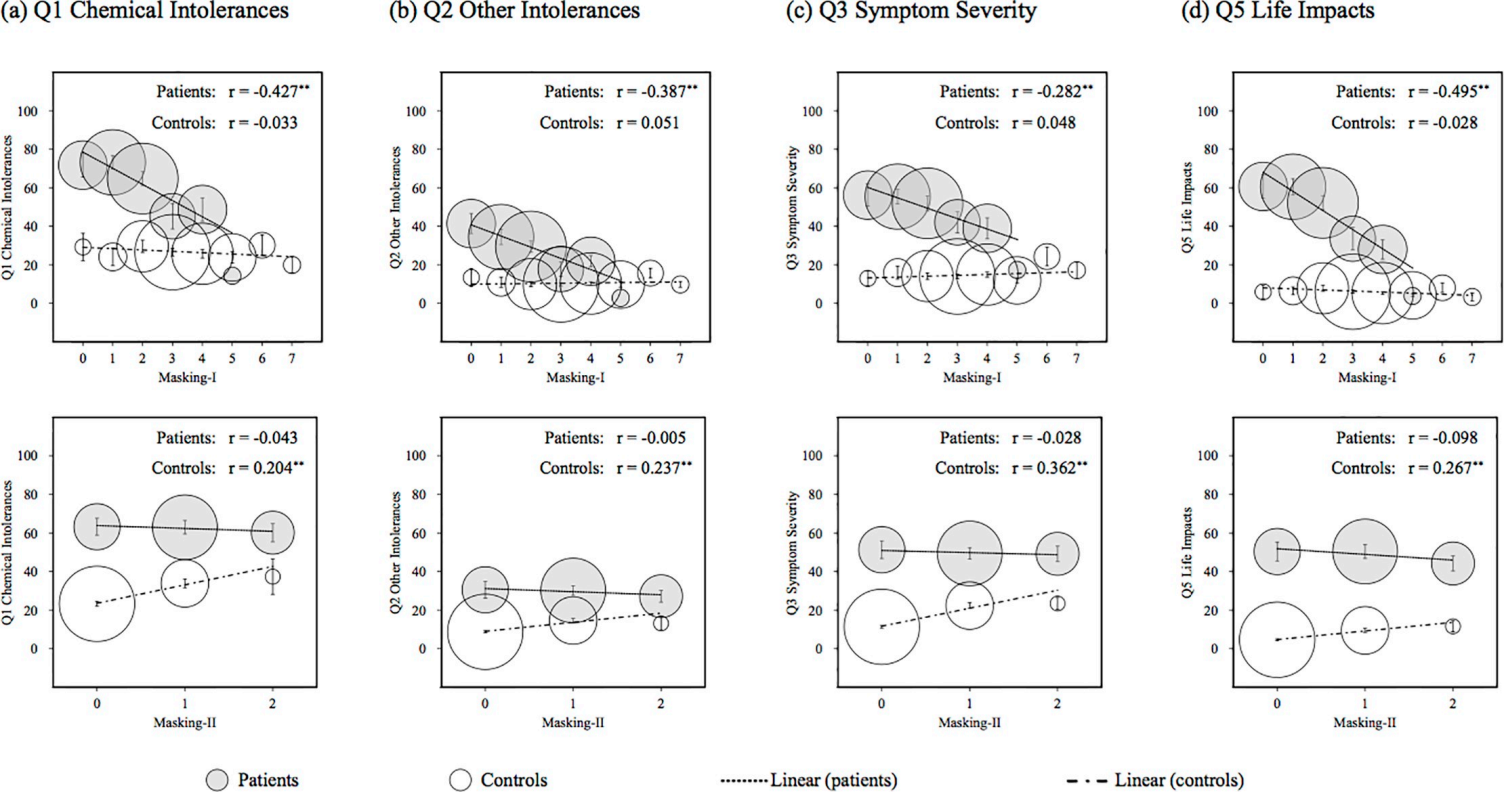
Scatter plots of the interaction between Masking-I or Masking-II and the other four scales in QEESI. Error bars indicate standard errors. The area of each circle indicates the proportion of participant numbers. The r is Spearman’s correlation coefficient and ** indicates *p* < 0.01. QEESI, Quick Environmental Exposure Sensitivity Inventory.

We divided the items of the Q4 Masking Index, which indicated a significant difference between patients and controls, into two scales. One was Masking-I, which was the total score of seven items with an odds ratio of less than 1 (q4.1, q4.2, q4.3, q4.4, q4.7, q4.8, and q4.9) and the other was Masking-II, which was the total score of two items with an odds ratio of more than 1 (q4.6 and q4.10). Fig 3 shows scatter plots between total scores for Masking-I or Masking-II and the other four scales.

#### Correlation to Masking-I

In terms of Masking-I, a significant negative correlation with each subscale total score was observed for patients, though a significant correlation was not observed for controls (Fig 3).

#### Correlation to Masking-II

In terms of Masking-II, a significant positive correlation with each subscale total score was observed for controls, though a significant correlation was not observed for patients, contrary to Masking-I (Fig 3).

### Logistic regression analysis

Table 5 shows the odds ratios of patients to controls for subscale total scores by binary and multiple logistic regression analyses. From the results of the binary logistic regression, of the four subscale total scores, the highest odds ratio was Q5 Life Impacts (1.126), followed in order by Q3 Symptom Severity (1.096), Q2 Other Intolerances (1.096), and Q1 Chemical Intolerances (1.054). Meanwhile, multiple regression analysis (Backward elimination [Wald]) was conducted using the covariates of Q1 Chemical Intolerances, Q2 Other Intolerances, Q3 Symptom Severity, Q5 Life Impacts, Masking-I, Masking-II, and the interaction of Q1 Chemical Intolerances and Masking-I. The interaction of Q1 Chemical Intolerances and Masking-II was excluded from the covariate because multicollinearity was observed between this and Masking-II. From the result of the multiple regression, among the four subscale total scores, the highest odds ratio was Q5 Life Impacts (1.085), followed in order by Q3 Symptom Severity (1.039), and Chemical Intolerances (1.036).

**Table 5:** Odds ratios of patients to controls for subscale total scores by binary and multiple logistic regression analyses and area under the ROC curve.

	Odds ratio one-point increase (95% CI)	*P*-value	Area under ROC curve (95% CI)
**Single scale**			
Q1 Chemical Intolerances	1.054 (1.044–1.065)	<0.001***	0.842 (0.800–0.883)
Q2 Other Intolerances	1.096 (1.075–1.117)	<0.001***	0.821 (0.777–0.865)
Q3 Symptom Severity	1.096 (1.079–1.114)	<0.001***	0.904 (0.872–0.936)
Q5 Life Impacts	1.126 (1.102–1.150)	<0.001***	0.946 (0.920–0.972)
Q4 Masking Index (total) (rev.)^a^	1.322 (1.163–1.503)	<0.001***	0.629 (0.567–0.690)
Masking-I (rev.)^a^	2.274 (1.883–2.746)	<0.001***	0.771 (0.721–0.822)
Masking-II	4.797 (3.353–6.864)	<0.001***	0.742 (0.688–0.796)
**Multiple scales**^**b**^			
Q1 Chemical Intolerances	1.036 (1.011–1.062)	0.005**	0.961 (0.942–0.979)
Q2 Other Intolerances	0.966 (0.928–1.005)	0.089	
Q3 Symptom Severity	1.039 (1.013–1.066)	0.003**	
Q5 Life Impacts	1.085 (1.057–1.113)	<0.001***	
Masking-II	2.700 (1.537–4.744)	<0.001***	
Q1 Chemical Intolerances × Masking-I	0.991 (0.984–0.998)	0.007**	

^a^Scores were reversed.

^b^Excluded variables; Masking-I

Wald test; significant at **p < 0.01, ***p < 0.001. ROC, receiver operating characteristic; CI, confidence interval; rev., reversed

The predicted probability (PrPr) was calculated using the equation:

$$PrPr=1/(1+e^{-R})$$ 1)

R=−4.691+0.035×Q1ChemicalIntolerances−0.035×Q2OtherIntolerances+0.038×Q3SymptomSeverity+0.993×Masking−II+0.081×Q5LifeImpacts−0.009×Q1ChemicalIntolerances×Masking−I 2)

where, *PrPr* is Predicted probability and *e* is the base of the logarithm. The remaining variables in the regression equation were suggested to be a reasonable scale for cut-off values and candidates for combined cut-off values.

### ROC analysis

**Fig 4:**
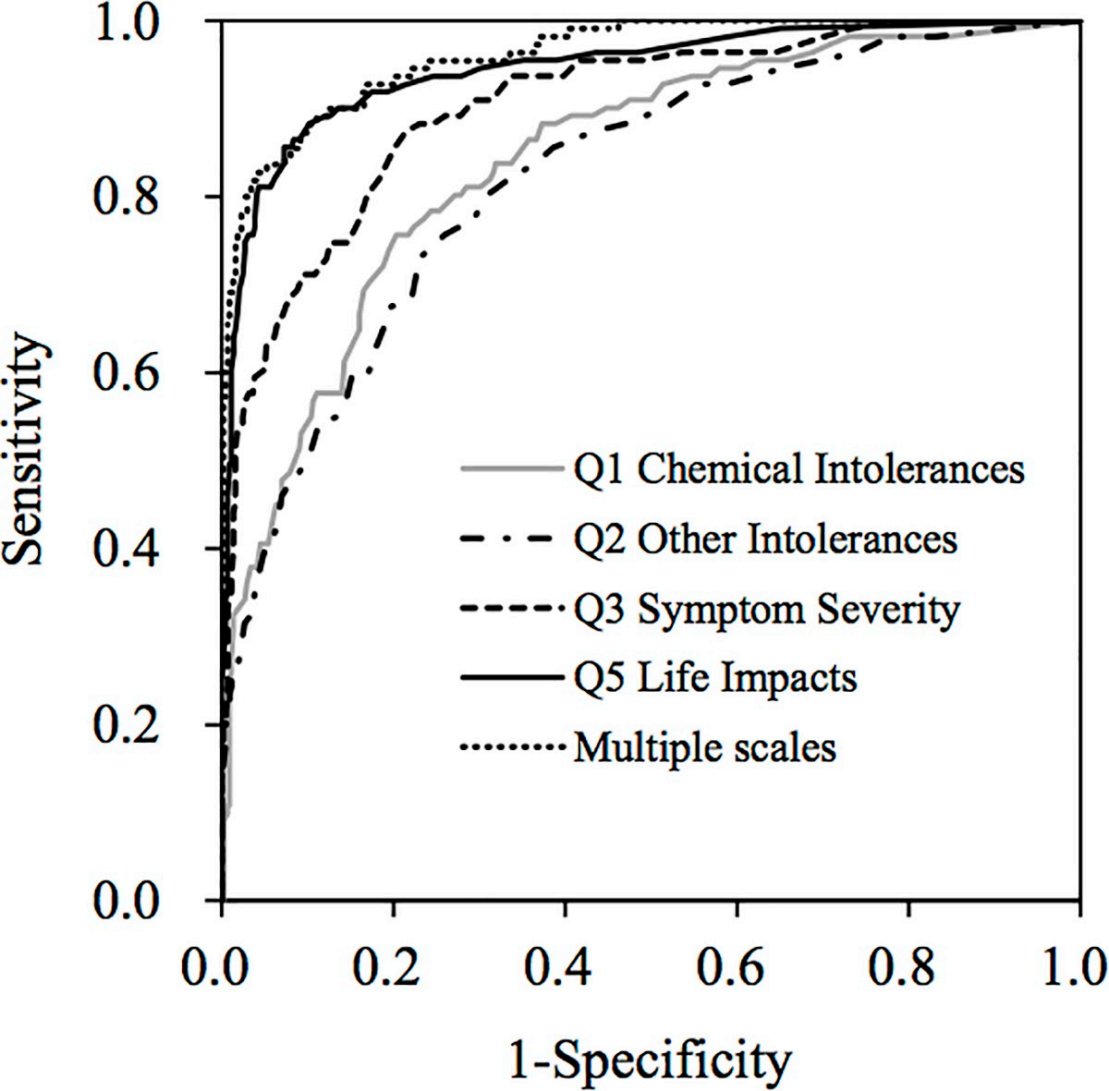
ROC curves for Q1 Chemical Intolerances, Q2 Other Intolerances, Q3 Symptom Severity, Q5 Life Impacts, and multiple scales. ROC, Receiver operating characteristic.

Table 5 shows the area under the ROC curve for single and multiple scales. ROC curves are shown in Fig 4. Regarding the area under the ROC for the four subscales, Q5 Life Impacts (0.946) was greatest, followed in order by Q3 Symptom Severity (0.904), Q1 Chemical Intolerances (0.842), and Q2 Other Intolerances (0.821). The area under the ROC for multiple scales was greater than those for single scales. For subscales relating to the Q4 Masking Index, the greater area under the ROC curve was for reversed Masking-I (0.771), followed in order by Masking-II (0.742), and reversed Q4 Masking Index (total; 0.629; Table 5). These results also suggested that it is better to divide the Q4 Masking Index into Masking-I and Masking-II and to consider the interaction with other subscales, than to evaluate the Q4 Masking Index as a total score of 10 items. The area under the ROC curve of multiple scales increased to 0.961 compared to a single scale.

### Setting new combined cut-off values

Table 6 shows the cut-off values for each single scale when the sum of sensitivity and specificity was highest. For the single scales, the sum of sensitivity and specificity was highest for Q5 Life Impacts (≥ 22, 178.3%), followed in order by Q3 Symptom Severity (≥ 25, 165.9%), Q1 Chemical Intolerances (≥48, 155.3%), and Q2 Other Intolerances (≥17, 150.0%). When using these three scales, the criteria meeting all conditions of Q1 Chemical Intolerances ≥ 30, Q3 Symptom Severity ≥ 17, and Q5 Life Impacts ≥ 13 indicated a high sum for sensitivity and specificity (175.7%). This value was high or comparable when compared to those obtained by applying our previous cut-off values or the cut-off values in foreign countries to data in this study (Table 6).

**Table 6:** Sensitivity and specificity of new cut-off values in this and previous studies.

	Cut-off value	Sensitivity (%)	Specificity (%)
**Single scale**			
Q1 Chemical Intolerances	48	75.7	79.6
Q2 Other Intolerances	17	73.0	77.0
Q3 Symptom Severity	25	87.4	78.5
Q5 Life Impacts	22	85.6	92.7
**Combined scales**			
Q1 Chemical Intolerances	30	82.0	94.4
Q3 Symptom Severity	13		
Q5 Life Impacts	17		
**Other cut-off values**			
Hojo et al., 2009^a^		90.1	75.9
Miller and Prihoda, 1999^b^		44.1	97.7
Skovbjerg et al., 2012^c^		80.2	92.6

^a^Met more than two of the three cut-off values of Q1 Chemical Intolerances ≥ 40, Q3 Symptom Severity ≥ 20, and Q5 Life Impacts ≥ 10

^b^Met all cut-off values of Q1 Chemical Intolerances ≥ 40, Q2 Other Intolerances ≥ 25, and Q3 Symptom Severity ≥ 40

^c^Met all cut-off values of Q1 Chemical Intolerances ≥ 35, and Q5 Life Impacts ≥ 14

### Classification according to risk for MCS using new combined cut-off values

We classified the 111 patients and 444 age- and gender-matched controls into four categories using the new criteria and Masking-I in this study using the same classification method as Miller & Prihoda [30] (Table 7). As a result, 91 patients (82.0%) were classified as Very suggestive, 5 (4.5%) were Somewhat suggestive, 12 (10.8%) were Problematic, and 3 (2.7%) were Not suggestive. Meanwhile, for controls, 25 (5.6%) were Very suggestive, 11 (2.5%) were Somewhat suggestive, 213 (48.0%) were Problematic, and 195 (43.9%) were Not suggestive.

**Table 7:** Distribution of patients classified according to risk of MCS using three cut-off values and Masking-I.

Risk of MCS	Q1 Chemical Intolerances	Q3 Symptom Severity	Q5 Life Impacts	Masking-I	MCS patients, n (%)		Controls, n (%)	
					16 categories	4 categories	16 categories	4 categories
Very suggestive	≥30	≥13	≥17	≥3	16 (14.4)	91 (82.0)	16 (3.6)	25 (5.6)
Very suggestive	≥30	≥13	≥17	<3	75 (67.6)		9 (2.0)	
Somewhat suggestive	<30	≥13	≥17	≥3	5 (4.5)	5 (4.5)	11 (2.5)	11 (2.5)
Problematic	<30	≥13	≥17	<3	0 (0.0)	12 (10.8)	3 (0.7)	213 (48.0)
Problematic	≥30	<13	≥17	≥3	1 (0.9)		2 (0.5)	
Problematic	≥30	<13	≥17	<3	0 (0.0)		2 (0.5)	
Problematic	≥30	≥13	<17	≥3	3 (2.7)		68 (15.3)	
Problematic	≥30	≥13	<17	<3	2 (1.8)		23 (5.2)	
Problematic	≥30	<13	<17	≥3	1 (0.9)		40 (9.0)	
Problematic	≥30	<13	<17	<3	0 (0.0)		12 (2.7)	
Problematic	<30	<13	≥17	≥3	1 (0.9)		1 (0.2)	
Problematic	<30	<13	≥17	<3	0 (0.0)		1 (0.2)	
Problematic	<30	≥13	<17	≥3	4 (3.6)		61 (13.7)	
Not suggestive	<30	≥13	<17	<3	1 (0.9)	3 (2.7)	11 (2.5)	195 (43.9)
Not suggestive	<30	<13	<17	≥3	1 (0.9)		144 (32.4)	
Not suggestive	<30	<13	<17	<3	1 (0.9)		40 (9.0)	

Very suggestive: study participants whose scores for Q1 Chemical Intolerances, Q3 Symptom Severity, and Q5 Life Impacts were higher than or equal to the cut-off values. Somewhat suggestive: study participants whose scores for Q3 Symptom Severity, Q5 Life Impacts, and Masking-I were higher than or equal to the cut-off values, and the Q1 Chemical Intolerances score was lower than the cut-off value. Not suggestive: study participants whose only Q3 Symptom Severity score among the four cut-off values was higher than or equal to the cut-off values or participants whose scores for Q1 Chemical Intolerances, Q3 Symptom Severity, and Q5 Life Impacts were lower than the cut-off values. Problematic: study participants who were not classified in the above three categories. MCS, Multiple chemical sensitivity

## Discussion

In this study, by considering new criteria for screening patients with MCS using QEESI based on the current situation in Japan, cut-off values with high sensitivity and specificity may be set as described below.

### Characteristics of patients with MCS

In considering the pathology of MCS, one important characteristic is that many patients are female. We have previously reported that more than 75% of MCS patients in Japan are women, a characteristic of this disease that is applicable worldwide [10–13]. One reason more females than males have MCS may be that females are more prone to hormonal fluctuations; female hormones are involved in the growth of hippocampal neural networks, with the hypothalamus associated with hippocampal circuits. Additionally, the pituitary–adrenal system is more sensitive in females than in males [51].

### Validity of new combined cut-off values

Only Q1 Chemical Intolerances included subscales of the question about sensitive reactions specific to MCS. Also, the subscales in Q3 Symptoms Severity and Q5 Life Impacts are not necessarily subscales specific to patients with MCS. Therefore, combined cut-off values are desirable that include Q1 Chemical Intolerances to screen patients with MCS from the general population. Q2 Other Intolerances could be excluded because Cronbach’s alpha, discriminant ability, and the area under the ROC curve were low or small, similar to that found in our previous studies [20, 22, 35]. It is reasonable to select three subscale total scores, namely Q1 Chemical Intolerances, Q3 Symptom Severity, and Q5 Life Impacts, as combined cut-off values. The sum of the sensitivity and specificity obtained using new combined cut-off values (Q1 Chemical Intolerances ≥ 30, Q3 Symptom Severity ≥ 17, and Q5 Life Impacts ≥ 13) was higher than those obtained when the cut-off values of our previous study [22] or other studies in the USA [30] and Denmark [27] were applied to recent data in this study (Table 6). In the frequency distributions shown in Fig 1, single and combined cut-off values are also indicated. Each mode value for patients and controls were contained separately on both sides of the new combined cut-off values, suggesting these values can discriminate controls from patients. As a result, it may be concluded that study participants who meet all conditions of Q1 Chemical Intolerances ≥ 30, Q3 Symptom Severity ≥ 17, and Q5 Life Impacts ≥ 13 can be screened as very subjective patients with MCS. These results highlight the validity of the new criteria in this study.

### Divided Masking Index and correlation to Q1 Chemical Intolerances

In this study, the Q4 Masking Index was divided into two scales: one consisted of seven items with an odds ratio of less than 1 (Masking-I) and the other consisted of two items with an odds ratio of more than 1 (Masking-II; Table 4).

It was presumed that patients with high scores (e.g. Q1, Q2, Q3, and Q5) avoided seven ongoing chemical exposures (e.g. tobacco, alcohol, caffeine, scented personal-care products, second-hand smoke, gas or propane stoves, and scented fabric softener) because these worsened their chemical intolerances and symptoms (Fig 3). These can be easily avoided as onset or symptom enhancement factors by patients with MCS in Japan.

In comparison, the two items of Masking-II may be regarded as factors that cannot easily be avoided. More specifically, “Chemical or smoke exposure in a job or hobby” may be the cause of the onset of MCS. Meanwhile, “Drugs” may be the cause of the onset of MCS, but drugs may have also been used to alleviate MCS symptoms (Fig 3). In addition, it is presumed that “q4.5 Insecticides” was not significantly different between patients and controls; they are not the only onset/trigger factors but are also items that cannot easily be avoided.

Moreover, the relationship between the divided Masking Index and Q1 Chemical Intolerances indicated a significant correlation for patients or controls (Fig 2), suggesting the existence of interactions between the divided Masking Index and Q1. This thus shows that it is necessary to divide the Masking Index into Masking-I and Masking-II and to consider the interaction with Q1 when screening patients with MCS using QEESI in Japan. For controls, a significant positive correlation was observed between Masking-II and Q1 Chemical Intolerances, suggesting the items of Masking-II were related to chemical intolerances, though the causal correlation was unknown. Meanwhile, for patients the higher the Masking-I score, the lower the score for Q1 Chemical Intolerances. As Miller and Prihoda [30, 31] have pointed out, this suggests two things: that patients’ intolerances were masked by everyday exposures and/or patients with a high chemical intolerance avoided chemical exposures. Therefore, when considering “Masking”, Masking-I may be reasonable instead of a Masking Index for screening patients with MCS in Japan.

### Classification according to risk of MCS

Miller and Prihoda [30] classified participants into four categories according to their risk of having a chemical sensitivity (Very suggestive, Somewhat suggestive, Problematic, and Not suggestive) by considering the Q4 Masking Index since people with high scores for masking items (ongoing chemical exposure) mask their symptoms. We also classified the 111 patients and 444 age- and gender-matched controls into four categories using the new criteria and Masking-I in this study using the same classification method as Miller & Prihoda [30] (Table 7). As a result, in total 96 patients (86.5%) were classified as Very suggestive (91 patients, 82.0%) or Somewhat suggestive (5 patients, 4.5%). Meanwhile, for controls, 25 controls (5.6%) were Very suggestive and 11 controls (2.5%) were Somewhat suggestive, and in total only 36 controls (8.1%) were classified as Very or Somewhat suggestive. As for the above results, it was shown that the new combined cut-off values with Masking-I are reasonable criteria to use in screening patients with MCS in Japan.

The controls in this study were not from a randomly selected general population and may therefore include selection bias. Azuma et al. [8] conducted a survey using QEESI targeted to 7,245 adults selected by stratified random sampling in Japan. These 7,245 adults were classified using new cut-off criteria divided into four categories. As a result, 456 participants (6.3%) were classed as Very suggestive, 188 participants (2.6%) were Somewhat suggestive, 3,470 participants (47.9%) were Problematic, and 3,130 participants (43.2%) were classed as Not suggestive. This distribution is quite similar to that of the controls in this study (Table 7). From this it can be concluded that the 444 controls in this study may be regarded as representing an average general population in Japan. Although none of the controls were diagnosed as having MCS, 5.6% were classified as Very suggestive and 2.5% were classified as Somewhat suggestive, meaning that potential patients with MCS made up approximately 6%, and people in the preliminary group approximately 3%, of adults in Japan.

Drs. Ishikawa, Miyata, Sakabe, and Mizuki, who are MCS specialist physicians in Japan, reported that many patients with MCS relieved their symptoms by appropriate treatments at an early stage; however, if left untreated, the MCS became severe, was harder to recover from, and having a normal life became impossible [45, 52]. Therefore, we strongly advise people classified as Very suggestive by the new criteria to consult a MCS specialist physician immediately. People classified as Somewhat suggestive may be “people in the preliminary group” who develop MCS by some trigger or are not aware of the symptoms of MCS. Thus, we advise that it is important to ensure such people become more aware of chemical exposures in their daily life.

However, Mizuki [45, 53] and Katoh [54] reported that the numbers of medical institutions that are equipped to deal with patients with MCS and physicians knowledgeable about MCS have been decreasing in Japan. Katoh further reports that "In Japan, the awareness of MCS is lower than that of other countries, so, many patients suffer from families and people around them who do not understand their illness." Therefore, considering that approximately 6% of suggestive patients with MCS in the Japanese population exist, there is an urgent need to increase the numbers of specialist physicians and physicians with an understanding of MCS in Japan. We think QEESI is an effective tool to educate physicians and medical personnel about MCS. In addition, to enlighten people in the preliminary group on the prevention of onset, the general public needs to be educated about MCS. QEESI and the new criteria in this study could be made available for these purposes.

### Allergic disease prevalence among patients with MCS

Patients with MCS had significantly higher rates of allergic diseases (e.g. bronchial asthma, allergic rhinitis, rash, hay fever, food allergies) compared to the controls (Table 1). These results are consistent with past reports [9, 13, 41, 50]. In addition, according to a survey using QEESI recently conducted in Korea, patients with MCS and allergic diseases compared with those without allergic diseases showed significantly higher scores for the four subscales of QEESI; in particular, the scores of patients with atopic dermatitis were significantly higher [41].

In response to a recent acute increase in the number of patients with MCS in Japan, six kinds of allergic diseases (bronchial asthma, atopic dermatitis, allergic rhinitis, allergic conjunctivitis, hay fever, and food allergies) have been targeted in basic law measures undertaken by the Ministry of Health, Labour and Welfare (Ministry of Health, Labour and Welfare, 2010; The House of Representatives, Japan, 2014). Hojo et al. [50] reported that of the prevalent allergic diseases among patients with MCS in Japan, bronchial asthma (adjusted odds ratio [AOR] of the new survey against the old survey: 5.19), atopic dermatitis (AOR: 3.77), allergic rhinitis (AOR: 5.34), and food allergies (AOR: 2.63) increased significantly, while hay fever (AOR: 0.38) and drug allergies (AOR: 0.40) decreased significantly compared to 10 years ago.

Because the prevalence of allergic diseases varies widely according to country, country-specific surveys and measures are indispensable. We think that QEESI is effective as a tool for analyzing the relationship between allergic diseases and MCS. We would therefore like to undertake further studies on the relationship between allergies and MCS symptoms for each allergic disease, particularly in Japan.

### Limitations of study and future challenges

The limitations of this study included the following: (1) The control group was not a randomly sampled general population. However, as described above, the results of classifying 7,245 randomly-sampled people from a general population into four categories in the study conducted by Azuma et al. [8] were almost in agreement with the classification results of the controls in this study, suggesting the latter could be presumed to be representative of a general population: (2) In this study, we did not examine differences in chemical intolerances and symptoms according to gender and age, which will be undertaken in future studies. Notably, more than 70% of patients with MCS are females and many patients are middle-aged [1, 12, 18, 21, 24, 29, 39, 50].

Regarding ongoing chemical exposure, only 10 items present in the Q4 Masking Index of QEESI were analyzed in this study. In Japan, adding new chemicals with significant health risks to the guidelines for the 13 indoor air pollutants mentioned above and revising the existing guidelines is presently being considered [55]. Consequently, it is necessary that studies be conducted after the inclusion of such new chemicals. We are aware that subjective symptoms of patients with MCS are relatively undefined and may be based on psychosomatic correlations such as mental illness and a nocebo effect [42, 44, 48, 56, 57]. Therefore, an effective and objective test method needs to be developed as a matter of urgency.

## Conclusions

We have set new combined cut-off values (Chemical Intolerances ≥ 30, Symptom Severity ≥ 13, and Life Impacts ≥ 17) with high sensitivity and specificity, which were based on current Japanese conditions. These new criteria should be used for screening and as diagnostic aids for Japanese patients with MCS. The use of these criteria and a divided Masking Index suggests that approximately 6% of very suggestive people with MCS and approximately 2.5% of somewhat suggestive people with MCS currently exist in Japan.

## Abbreviations

QEESI: Quick Environmental Exposure Sensitivity Inventory
MCS: Multiple Chemical Sensitivity
Q1: Chemical Intolerances
Q2: Other Intolerances
Q3: Symptom Severity
Q5: Life Impacts
